# Glucocorticoid reduction after starting crinecerfont in adult patients with classic CAH: practical perspectives

**DOI:** 10.1210/clinem/dgag147

**Published:** 2026-04-01

**Authors:** Oksana Hamidi, Richard J Auchus, Ricardo Correa, Alice C Levine, Margaret E Wierman, Andrea L Hartzell, Vivian H Lin, Irina Bancos

**Affiliations:** Division of Endocrinology, Department of Internal Medicine, University of Texas Southwestern Medical Center, Dallas, TX 75390, USA; Division of Metabolism, Endocrinology, and Diabetes, Departments of Internal Medicine and Pharmacology, University of Michigan Medical School, Ann Arbor, MI 48109, USA; Endocrinology and Metabolism Section, Medicine Service, LTC Charles S. Kettles Veterans Affairs Medical Center, Ann Arbor, MI 48105, USA; Department of Endocrinology, Medical Specialty Institute, Cleveland Clinic, Cleveland, OH 44195, USA; Division of Endocrinology, Diabetes, and Bone Diseases, Department of Medicine, Icahn School of Medicine at Mount Sinai, New York, NY 10029, USA; Departments of Medicine, Integrative Physiology, and OBGYN, University of Colorado Anschutz Medical Campus, Aurora, CO 80045, USA; Medical Affairs, Neurocrine Biosciences, San Diego, CA 92130, USA; Medical Affairs, Neurocrine Biosciences, San Diego, CA 92130, USA; Division of Endocrinology, Diabetes, Metabolism, and Nutrition, Department of Internal Medicine, Mayo Clinic, Rochester, MN 55905, USA

**Keywords:** 17-hydroxyprogesterone, 21-hydroxylase deficiency, androstenedione, congenital adrenal hyperplasia, crinecerfont, glucocorticoid

## Abstract

**Context:**

Crinecerfont, a first-in-class corticotropin-releasing factor type 1 receptor antagonist, is FDA-approved as an adjunct to the physiologic glucocorticoid (GC) required to treat the underlying adrenal insufficiency in patients (≥4 years old) with classic congenital adrenal hyperplasia (CAH). By reducing elevated adrenocorticotropic hormone, crinecerfont controls excess adrenal androgens, thereby enabling lower GC doses. However, there are no published recommendations for how to reduce supraphysiologic GC doses after initiating crinecerfont. This manuscript aims to provide a framework developed by experienced endocrinologists for reducing supraphysiologic GC doses in patients taking crinecerfont.

**Evidence Acquisition:**

An experienced panel of 11 endocrinologists discussed strategies and considerations when reducing GC doses and developed an algorithm to guide GC dose reductions after introducing crinecerfont in adults with CAH.

**Evidence Synthesis:**

Approaches to GC reduction should be individualized based on the patient's therapeutic goals, cortisol needs, lifestyle preferences, and the clinician's experience to set appropriate targets for clinical parameters, androgens, and GC dose regimen. In general, GC doses should be reduced gradually to minimize the risks of developing symptoms of GC withdrawal or adrenal insufficiency. GC doses should be adjusted to the lowest level needed to maintain androgens at goal, without reducing below what is needed for cortisol replacement. Practical considerations during GC dose reduction include switching from longer- to shorter-acting GCs (eg, from dexamethasone to hydrocortisone), optimizing dose distribution to mimic normal circadian cortisol exposure, and consolidating or eliminating doses to improve adherence.

**Conclusion:**

This framework for reducing supraphysiologic GC doses in adult patients taking crinecerfont may become increasingly relevant as treatment of CAH shifts toward physiologic GC replacement with adjunctive control of adrenal androgens.

Congenital adrenal hyperplasia (CAH), a rare inherited condition characterized by inadequate synthesis of cortisol and often aldosterone (1-3), can be caused by a deficiency in any of the enzymes or cofactor proteins required for cortisol biosynthesis, and is most commonly due to 21-hydroxylase deficiency (21-OHD; ∼95% of cases) (1-5). Without cortisol to provide negative feedback on the hypothalamic–pituitary–adrenal (HPA) axis, there is increased secretion of hypothalamic corticotropin-releasing factor (CRF) and pituitary adrenocorticotropic hormone (ACTH) (6, 7). The excess ACTH leads to elevated production of steroid precursors in the adrenal glands, which are then shunted down the unaffected androgen synthesis pathways, resulting in excess adrenal androgen production (1, 2, 8).

The phenotype of 21-OHD is a continuum based on the degree of residual enzyme activity, and is generally categorized into 3 forms: salt-wasting, simple virilizing, and nonclassic (3, 4, 7). In the most severe, salt-wasting classic form, there is little to no enzyme activity, resulting in severe deficiency of both cortisol and mineralocorticoid (usually aldosterone) that can cause potentially fatal salt-wasting crises. The simple virilizing classic form is characterized by cortisol deficiency, but some aldosterone activity. The mildest form, nonclassic 21-OHD, results when there is decreased enzyme activity but sufficient cortisol production (3, 4, 7).

Other rare forms of classic CAH share many similarities with 21-OHD, including ACTH excess, cortisol deficiency, and, in some conditions (eg, 11β-hydroxylase deficiency), androgen excess. In this article, we will focus on classic 21-OHD (hereafter referred to as “CAH”), including both salt-wasting and simple virilizing forms. The general principles of management and glucocorticoid (GC) dose reduction also apply to the rare forms, although the specific laboratory tests, mineralocorticoid requirements, and treatment targets might differ among these various enzyme deficiencies.

## Disease-related consequences of CAH

Primary adrenal insufficiency can cause multiple symptoms, including fatigue, lethargy, muscle weakness, decreased appetite and unintentional weight loss, dizziness upon standing, and hypotension (9, 10). When adrenal insufficiency is severe, as in times of acute intercurrent illness or physiologic stress, all patients with CAH are at risk of life-threatening adrenal crisis (see ***Adrenal Crisis***) (1, 11-13). Patients with salt-wasting CAH are especially at risk of adrenal crisis, with potential for more severe presentation due to sodium and water loss caused by mineralocorticoid insufficiency (11, 14).

**Figure 1:**
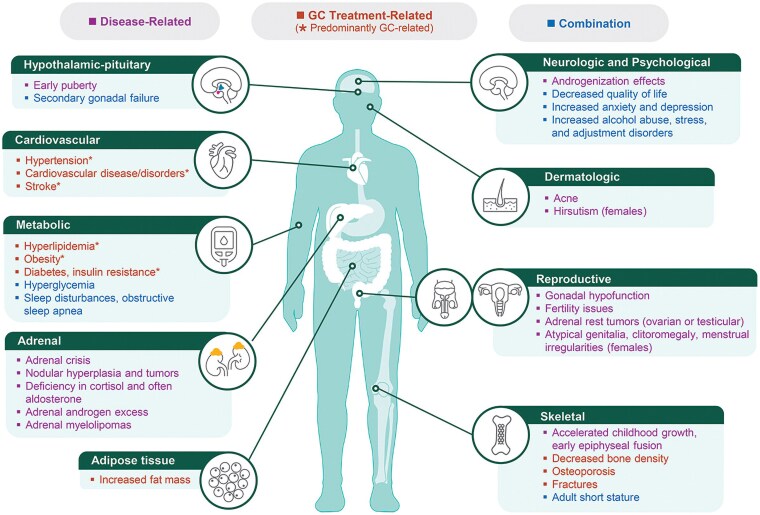
Conditions associated with CAH. Reproduced from Bancos I et al: Glucocorticoid therapy in classic congenital adrenal hyperplasia: traditional and new treatment paradigms. *Expert Rev Endocrinol Metab.* 2025;20(1):33-49 (10), by permission of the publisher Informa UK Ltd t/a Taylor & Francis Ltd. © 2025 Taylor & Francis Group. Originally from Merke D and Auchus R, *N Engl J Med* 2020 (1) (© 2020 Massachusetts Medical Society), adapted in Bancos 2025 with permission from the Massachusetts Medical Society. Abbreviations: CAH, congenital adrenal hyperplasia; GC, glucocorticoid.

Patients with CAH can also experience the consequences of excess ACTH and androgen production (Fig. 1). Beginning in utero and continuing through adulthood, excess androgens cause virilization in female patients (7, 15, 16). In both male and female patients, continued exposure to excess androgens during childhood causes early puberty and advanced bone age that can impact final adult height (1, 17-19). During adolescence and adulthood, excess ACTH and/or androgens can lead to hirsutism, acne, menstrual irregularities, and rarely, development of ovarian adrenal rest tumors in female patients (7, 20-23). Male patients can develop testicular adrenal rest tumors (TARTs) due to excess ACTH, and excess androgens can cause secondary hypogonadism (7, 24-28). During adulthood, androgen excess can affect fertility in both male and female patients (29-32). In addition, adults with CAH are at increased risk for cardiovascular and metabolic problems such as obesity and insulin resistance, predominantly due to chronic supraphysiologic GC therapy (discussed later) but also to excess androgens (8, 25, 33-36).

## Glucocorticoid treatment for CAH

Patients with CAH need lifelong GC therapy to compensate for cortisol deficiency (1, 2, 10). In addition to cortisol replacement, GC treatment also suppresses excess ACTH and androgens. However, using GCs alone to manage ACTH and androgen levels often requires supraphysiologic doses (ie, higher than the mean daily cortisol production rate of 6 to 8 mg/m^2^/d or ∼10 to 15 mg/d [range, 2 to 14 mg/m^2^/d or 5 to 25 mg/d] in healthy individuals (37-39)), longer-acting GCs, and/or dosing at nonphysiologic times (8, 40, 41). Long-term exposure to supraphysiologic GCs can lead to adverse cardiovascular, metabolic, and skeletal outcomes (Fig. 1) (3, 8, 28, 34, 36, 42-64).

The increased risks of developing cardiometabolic comorbidities such as hypertension, cardiovascular disease, obesity, insulin resistance, and diabetes mellitus have been well documented in patients with CAH, especially those receiving higher GC doses (34, 36, 42-45, 57-61). Of the common GCs used in CAH, dexamethasone is generally associated with the most negative impacts on cardiometabolic health; patients taking dexamethasone have higher body mass index (BMI), increased waist circumference and fat mass, and insulin resistance compared to those taking hydrocortisone (36, 58, 62-64).

Long-term use of GCs, especially at higher doses, can also increase bone resorption and inhibit bone formation, leading to increased risk of low bone mineral density (BMD), osteoporosis, and fractures (3, 46-49). Several smaller studies have shown associations between higher GC dose and reduced BMD or BMD *z* scores in patients with CAH (50-54). Furthermore, lower BMD has been reported in patients on synthetic, longer-acting GCs (eg, dexamethasone and prednisolone) compared to patients on hydrocortisone (55, 56).

## New approaches to CAH

Emerging strategies in GC therapy aim to replicate the physiologic pharmacokinetics of endogenous cortisol more closely. Strategies to optimize GC delivery in CAH include continuous subcutaneous hydrocortisone infusion (CSHI) and modified-release hydrocortisone formulations (65-69). While CSHI remains unlicensed, 2 modified-release hydrocortisone formulations are approved in Europe—Plenadren® for adrenal insufficiency and Efmody® for CAH.

Several new treatment strategies are being investigated to reduce ACTH-mediated adrenal androgen production. These non-GC treatment strategies would not eliminate the need for physiologic cortisol replacement, but potentially allow for lower, more physiologic GC doses when added to the patient's GC treatment (7, 10, 70). Crinecerfont is a first-in-class CRF type 1 receptor (CRF_1_) antagonist that is FDA-approved as an adjunct to the physiologic GC required to treat the underlying adrenal insufficiency in CAH. By reducing elevated ACTH, crinecerfont controls adrenal androgens in both the salt-wasting and simple virilizing forms of CAH (70-76). Other non-GC therapies currently under investigation for the treatment of CAH, including Lu AG13909 (an anti-ACTH monoclonal antibody) and atumelnant (a melanocortin type 2 receptor antagonist), are being evaluated in clinical trials (70, 77, 78). In contrast, development of tildacerfont (another CRF_1_ antagonist) and BBP-631 (a gene therapy candidate) were terminated in 2024; and that of nevanimibe (a sterol-*O*-acyltransferase 1 inhibitor) in 2020 (70, 79).

**Figure 2:**
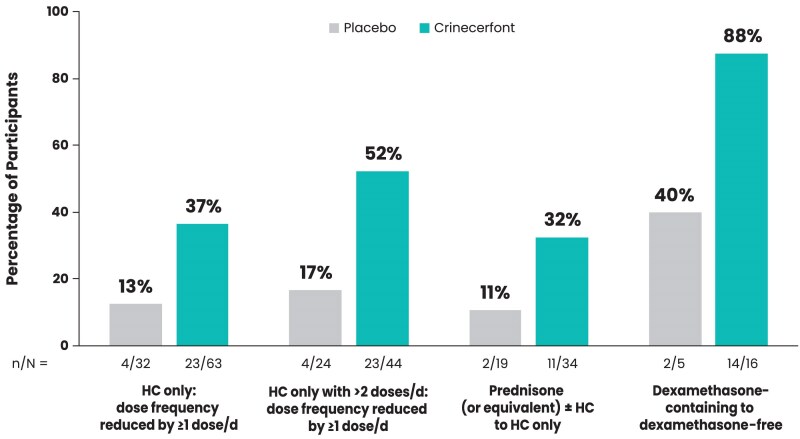
Achievement of reduction in treatment burden or nonphysiologic GCs. Abbreviations: GC, glucocorticoid; HC, hydrocortisone.

In 2 open-label phase 2 clinical trials in patients with CAH, crinecerfont reduced elevated ACTH, 17-hydroxyprogesterone (17-OHP), and androstenedione (71, 72). In phase 3 studies, significant decreases in these hormones were observed with crinecerfont compared to placebo (73-76). These decreases allowed for clinically meaningful reductions in GC dose with crinecerfont vs placebo while androstenedione levels were maintained or improved (73, 75). Investigators were advised to transition patients to a less potent GC, reduce dosing frequency where feasible, and reduce or discontinue non-physiologic dosing regimens (eg, bedtime GC administration); more patients on crinecerfont were able to sustain reductions in total GC dose, decreases in hydrocortisone dosing frequency, and/or switch to a more physiologic GC regimen (eg, predniso[lo]ne-containing regimen to hydrocortisone alone, or dexamethasone-containing to dexamethasone-free regimen) (Fig. 2).

During the double-blind period of the phase 3 study in adult patients, GCs were reduced every 2 weeks in 1 to 4 steps, regardless of androstenedione levels, to a target of 8 to 10 mg/m^2^/d hydrocortisone equivalents (HCe). However, this rapid and fixed reduction schedule is often not appropriate in clinical settings where patients are not monitored as frequently as in a clinical trial, and many patients will better tolerate a more gradual reduction. As there have been no published recommendations for reducing GC doses after initiating crinecerfont, we sought to provide a general framework for how to approach GC dose reductions in patients taking crinecerfont.

## Development of recommendations for approach to GC dose reductions after initiating crinecerfont

**Figure 3:**
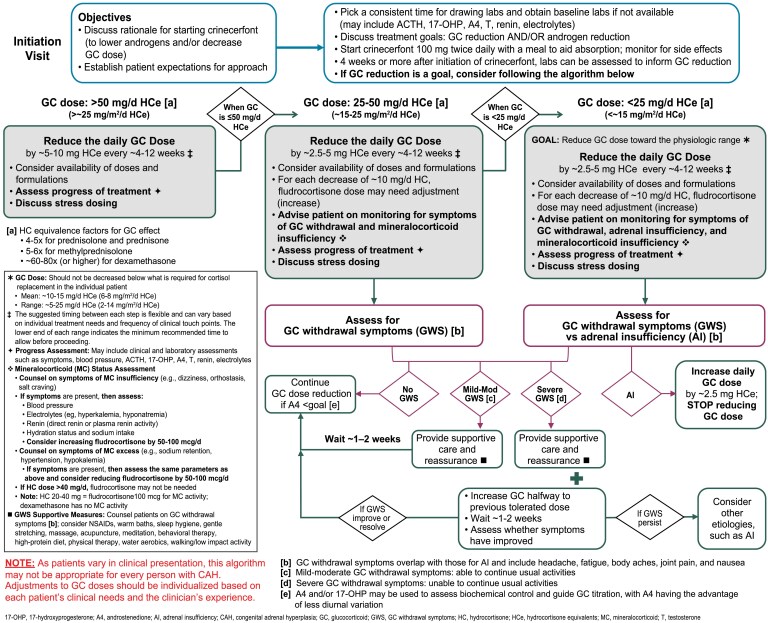
Approach to GC reduction with initiation of crinecerfont in adult patients (age ≥18 years) with classic CAH: practical perspectives. Abbreviations: 17-OHP, 17-hydroxyprogesterone; A4, androstenedione; ACTH, adrenocorticotropic hormone; AI, adrenal insufficiency; CAH, congenital adrenal hyperplasia; GC, glucocorticoid; GWS, GC withdrawal symptoms; HC, hydrocortisone; HCe, hydrocortisone equivalents; MC, mineralocorticoid; NSAIDs, nonsteroidal anti-inflammatory drugs; T, testosterone.

Eleven endocrinologists (5 adult, 6 pediatric) with extensive experience treating and managing patients with CAH were invited by Neurocrine Biosciences, Inc. (San Diego, CA) to participate in a panel on crinecerfont and GC dose reduction. In December 2024, they provided input on overall strategy and key considerations when reducing GC doses in patients with CAH. A smaller group of 2 adult endocrinologists and 3 pediatric endocrinologists re-convened within a month to review the previous discussions and develop recommendations for adult and pediatric patients, respectively. All authors individually reviewed and provided feedback on these recommendations and agreed on the final algorithm (Fig. 3). Recommendations for pediatric patients are reported separately.

## Initial visit

Prior to initiating treatment with crinecerfont, it is important to discuss the patient's expectations and treatment goals, as well as their rationale for starting crinecerfont: to facilitate androgen reduction, reduce GC dose, change GC type, and/or simplify GC regimen. Given the well-documented negative effects of long-term exposure to GCs and that an empirically determined daily GC replacement dose can range from 2 to 14 mg/m^2^/d in adults with adrenal insufficiency (37), many patients with CAH can benefit from reducing their GC dose, especially those with signs of GC overtreatment (eg, suppressed androstenedione or 17-OHP, exogenous Cushing syndrome, metabolic comorbidities, decreased bone density or osteoporosis). Other patients with CAH may prioritize reducing ACTH and androgen levels in order to decrease the risk of developing adrenal rest tumors and preserve fertility, and/or to reduce clinical symptoms of hyperandrogenism (eg, hirsutism, acne, irregular menstruation). Based on the goals and presentation, the patient and clinician can set appropriate targets for clinical parameters, androgens, and GC dose regimen.

Before starting crinecerfont, we recommend obtaining baseline laboratory and clinical assessments. Laboratory assessment of androgen control may include measurements of 17-OHP, androstenedione, and testosterone. In addition, renin (direct renin or plasma renin activity), electrolytes, and blood pressure should be measured to assess for mineralocorticoid excess or insufficiency. Labs can be obtained before or after the morning GC dose (eg, 2–3 hours post dose), but it is important for the clinician and patient to decide on a consistent time of day and timing relative to the last GC dose for lab draws in order to set appropriate target goals and make meaningful comparisons across visits. Androstenedione levels, for example, will be highest in the morning before the first GC dose, and will be significantly lower post-GC dose. For patients pursuing fertility, tighter androgen (and progesterone) targets are often preferred, making pre-GC dose measurements more informative. In contrast, for those prioritizing metabolic health, less stringent androgen goals are appropriate, and post-GC dose values may be sufficient to guide therapy.

In addition to laboratory assessments, all patients should have height and weight recorded to calculate BMI and body surface area (BSA). Women should be assessed for hirsutism and menstrual regularity. Men should be assessed for testicular atrophy, TARTs, and testicular function—for example, with physical exam, scrotal ultrasound, gonadotropin measurement, and semen analysis when relevant. In addition, a ratio of androstenedione-to-testosterone <0.5 (calculated using the same units of measurement for both androstenedione and testosterone, eg, ng/dL or nmol/L) can indicate that appropriate proportions of hormones are produced by the gonads compared to the adrenal gland.

At the initial visit, patients should be informed about relevant prescribing information for crinecerfont, such as the recommended adult dosage of 100 mg orally, taken twice daily in the morning and evening with a meal or snack. There is no specific caloric or macronutrient requirement for this meal, but it should contain some fat. Five grams of fat (eg, a glass of whole milk, 1 large egg, or a tablespoon [15 mL] of peanut butter) would be sufficient to aid absorption. In addition, patients should be advised of the commonly reported adverse reactions in adults (Table 1) (80). There were few incidents of acute adrenal insufficiency or adrenal crisis (1.6% with crinecerfont; 0% with placebo) and no deaths in the double-blind, placebo-controlled trial (73, 80); however, it is important to counsel patients of the risk of life-threatening adrenal crisis with inadequate GC therapy. In patients taking a concomitant moderate cytochrome P450 3A4 (CYP3A4) inducer, the evening crinecerfont dose should be increased to 200 mg; the recommended dose for patients taking a concomitant strong CYP3A4 inducer is 200 mg in the morning and evening.

**Table 1:** Adverse reactions in adults with classic CAH treated with crinecerfont

Adverse Reactions*^a^*	Crinecerfont (N = 122) %	Placebo (N = 59) %
Fatigue	25	15
Headache	16	15
Dizziness	8	3
Arthralgia	7	0
Back pain	6	3
Decreased appetite	4	2
Myalgia*^b^*	4	3

^*a*^Occurring at a rate of ≥4% in the crinecerfont group and more frequently than in placebo (80).

^*b*^Includes the MedDRA preferred terms: muscle spasms, muscle tightness, musculoskeletal discomfort, and myalgia.

Abbreviations: CAH, congenital adrenal hyperplasia; MedDRA, Medical Dictionary for Regulatory Activities.

Before initiating GC dose reduction, patients should be advised of the potential for GC withdrawal symptoms, which can occur in any individual undergoing GC tapering. These symptoms are more common in those previously treated with higher GC doses or with potent, long-acting agents such as dexamethasone, and especially if the dose is reduced too quickly (see ***GC Withdrawal***) (81). Given the well-established risk of Cushingoid toxicity associated with dexamethasone—even at relatively low doses due to its high potency and prolonged half-life—long-term use should generally be avoided when possible. Transitioning to a shorter-acting GC such as hydrocortisone or predniso[lo]ne should therefore be considered a treatment priority for most patients. If a change from dexamethasone is planned, it is reasonable to implement this switch first and manage any withdrawal or dose-adjustment effects before adding crinecerfont. Although conversion to a shorter-acting GC may require more frequent dosing, this approach typically reduces cumulative toxicity and improves physiologic GC exposure (7, 8, 36, 64). As evidenced in the CAHtalyst® Adult clinical trial (73), the switch from dexamethasone to a shorter-acting GC can also be done after initiating crinecerfont—in which case the timing of the twice-daily crinecerfont administration can be coordinated with GC administration if appropriate.

Patients should also be educated on GC stress dosing and monitoring for symptoms of adrenal insufficiency prior to any GC reductions (see ***Stress Dosing*** and ***Adrenal Insufficiency***). In addition, it is critical to emphasize the risk of life-threatening adrenal crisis if they stop taking their GC medication or do not take enough GC, especially in a situation of increased cortisol need (see ***Adrenal Crisis***). Although non-GC therapies such as crinecerfont can reduce excess ACTH and/or androgens, thereby allowing for lower GC doses, GC treatment is still required for cortisol replacement and should never be discontinued.

## Setting goal GC dose

To facilitate shared decision-making and ensure patient comfort with GC tapering, it may be helpful to draft a proposed GC type and dose-reduction plan in advance and review it with the patient. This approach provides a clear framework for transitioning to the target GC regimen—both in timing and dose—and may streamline the adjustment process by setting expectations and promoting collaborative care.

GC doses should be adjusted to the lowest level that achieves target androgen levels while preserving adequate cortisol replacement to meet physiologic needs. Assessing physiologic GC replacement can be challenging, particularly in patients with CAH, where there can be substantial interindividual differences in cortisol production and BSA. BSA can be calculated using standard formulas such as the Du Bois formula: BSA (m^2^) = 0.007184×height (cm)^0.725^×weight (kg)^0.425^. The estimated mean daily cortisol production rate in healthy individuals is approximately 6 to 8 mg/m^2^/d, with a broader range of ∼2 to 14 mg/m^2^/d (37-39). When applied to an average adult BSA of 1.8 m^2^, this translates to a physiologic hydrocortisone dose of roughly 5 to 25 mg/d, assuming near-complete oral bioavailability.

**Figure 4:**
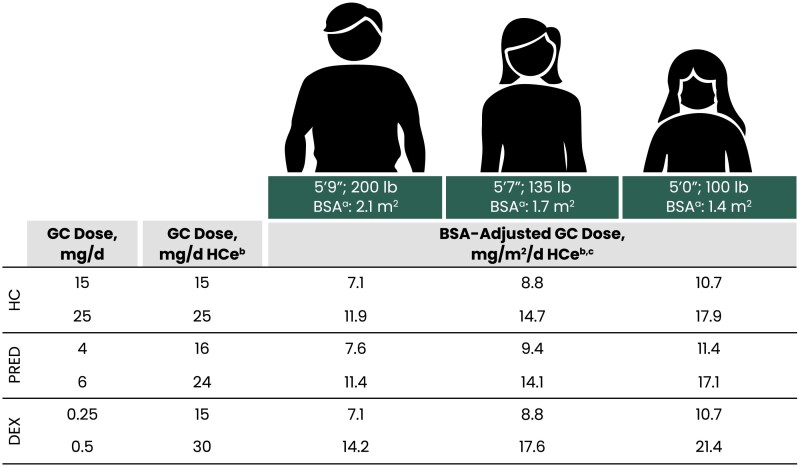
Examples of GC dose variation by body surface area. ^a^ Calculated using standard formulas such as the Du Bois formula: BSA (m^2^) = 0.007184×height (cm)^0.725^×weight (kg)^0.425^. ^b^Using the lower end of hydrocortisone equivalence factors as follows: 4 to 5x for prednisolone and prednisone; 60 to 80x for dexamethasone. ^c^The mean daily cortisol production rate is ∼10 to 15 mg/d or 6 to 8 mg/m^2^/d [range, 5 to 25 mg/d or 2 to 14 mg/m^2^/d] in healthy individuals. Abbreviations: BSA, body surface area; DEX, dexamethasone; GC, glucocorticoid; HC, hydrocortisone; HCe, hydrocortisone equivalents; PRED, prednisolone or prednisone.

As illustrated in Figure 4, there is no universal cutoff for a physiologic GC dose. Clinical judgment remains essential to determine whether a patient has reached an appropriate replacement level, taking into account symptoms, biochemical markers, and individual body size.

## Approach to GC dose reduction

**Figure 5:**
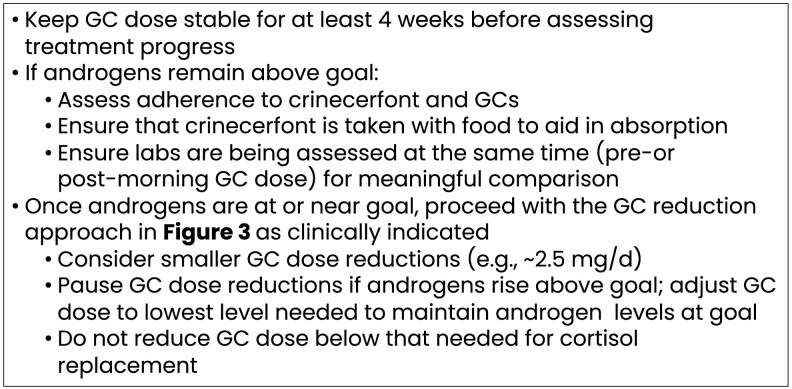
Considerations if androgens >goal before starting crinecerfont. Abbreviation: GC, glucocorticoid.

After initiating crinecerfont, laboratory measurements of androgen levels may be assessed ≥4 weeks later to inform the approach to GC reduction. Appropriate target levels for androgens vary from patient to patient depending on age, sex, individual treatment goals, clinical markers of disease control, and timing of laboratory assessments (2). Androstenedione and 17-OHP may be used to assess biochemical control and guide GC titration, with androstenedione having the advantage of less diurnal variation (2, 8). A general goal for androstenedione may be ∼1 to 1.5x the upper limit of normal for sex and age when measured before the morning GC dose, or within the normal range when measured after the morning GC dose. For 17-OHP, serum levels within the normal range may reflect excess GC exposure; rather, a serum level of ≤1200 ng/dL may be considered an acceptable target for many patients (2, 8, 82). For patients with androgens higher than the individual treatment goal, do not start to reduce GC dose until androgens have reached the target appropriate for the patient (see Fig. 5).

Once androgens are at goal, proceed with the GC reduction algorithm in Fig. 3 as clinically indicated. The algorithm is intended to provide guidance for reducing GC doses after starting crinecerfont; however, as patients vary in clinical presentation, it may not be appropriate for every adult patient with classic CAH. All GC dose adjustments should be individualized based on the patient's therapeutic goals, cortisol needs, and the clinician's experience.

As a first step—if this was not done prior to starting crinecerfont—consider switching from dexamethasone to hydrocortisone or prednisolone (or equivalent) (Table 2). Most patients will benefit from this switch due to the lower toxicity of shorter-acting GCs; long-term use of dexamethasone should generally be avoided if possible due to its negative effects on cardiometabolic and bone health (7, 8, 36, 64).

**Table 2:** Practical GC dosing optimization to improve adherence and outcomes*^a^*

Clinical consideration	Recommended action	Rationale	Notes for individualization
**GC type selection**	Switch from dexamethasone to hydrocortisone or predniso[lo]ne	Shorter-acting GCs have lower toxicity; dexamethasone is linked to adverse cardiometabolic and bone effects	Consider switching before initiating crinecerfont to be able to distinguish and address symptoms related to changing the GC type
**Non-physiologic evening dose**	Reduce or eliminate bedtime/evening GC dose	Mimics natural cortisol rhythm; avoids unnecessary suppression of morning ACTH and androgens	Especially relevant if crinecerfont is used, as it provides continuous CRF_1_ inhibition
**Dose distribution**	Take ∼2/3 of total daily GC dose in the morning; ∼1/3 in the afternoon	Aligns with circadian 24-hour cortisol profile	Adjust based on patient lifestyle and adherence needs
**Lifestyle-based tailoring**	Consider eliminating the afternoon dose for patients with busy schedules	Improves adherence in young adults or those with afternoon activities	Monitor for signs of under-replacement; some patients may need a stress dose for strenuous physical activity
**Habit stacking**	Align GC dosing with crinecerfont and meals/snacks	Enhances adherence through routine-building	Use patient's existing habits to anchor medication timing

^*a*^Immediate-release hydrocortisone generally should not be given less than twice daily.

Abbreviations: ACTH, adrenocorticotropic hormone; CRF_1_, corticotropin-releasing factor type 1 receptor; GC, glucocorticoid.

As a next step, consider reducing or eliminating the dose that is given at the most non-physiologic time (ie, bedtime or evening dose), as this may better mimic the 24-hour cortisol profile of an individual without underlying adrenal insufficiency (83, 84). The therapeutic regimen of crinecerfont is expected to maintain plasma exposures that provide continuous 24-hour CRF_1_ inhibition. This may eliminate the need for nonphysiologic evening doses often used in CAH to suppress morning ACTH and androgen production. This approach to arrive at circadian GC exposure was successfully used for the scheduled dose reduction process in the adult phase 3 crinecerfont trial (73). However, to reduce the risk of GC withdrawal symptoms, we generally recommend considering a slower down-titration process (eg, every 4 to 12 weeks) than the every-2-week reduction employed in the trial.

Ideally, GC treatment will approximate the 24-hour cortisol profile of an individual without adrenal insufficiency, with ∼2/3 of the total daily dose given in the morning and ∼1/3 in the afternoon. However, each patient's dose distribution should be tailored to best accommodate their lifestyle preferences and optimize adherence. For example, in a busy young adult taking ≥3 daily doses of immediate-release hydrocortisone, eliminating an afternoon dose could help improve adherence. In addition, decreasing to 2 daily GC doses and aligning the timing together with crinecerfont dosing and a meal or snack could help promote adherence through habit stacking (Table 2). Immediate-release hydrocortisone generally should not be given less than twice daily. Modified-release hydrocortisone formulations that delay peak cortisol exposure, though not currently available in the USA, can also be considered to shift the timing of GC doses and/or reduce the number of GC doses.

The recommended magnitude of individual dose reductions ranges from 2.5 to 10 mg/d HCe, depending on the starting dose, with ∼4 to 12 weeks between each dose reduction. Larger dose reductions are suggested with higher starting GC doses, and smaller reductions should be made when approaching physiologic GC doses. Each step should consider how well the patient tolerated previous GC reductions, as well as the GC potency (based on hydrocortisone equivalence factors: 4 to 5x for prednisolone and prednisone; 5 to 6x for methylprednisolone; 60 to 80x [or higher] for dexamethasone) (81, 85, 86) and the availability of dosage strengths and formulations.

Androgen levels should be monitored periodically during the GC dose reduction process. Laboratory values should be assessed at the same time (pre- or 2-3 hours post-morning GC dose) for each measurement to allow for meaningful comparison. If androgens begin to rise above target and/or the patient develops signs or symptoms of hyperandrogenism, consider pausing reductions in GC dose and assessing the patient's adherence to both GCs and crinecerfont, including whether crinecerfont is taken with a meal or snack to aid absorption. If androgen levels continue to rise, consider increasing the GC dose.

In addition, renin (direct renin or plasma renin activity) and volume status should be assessed regularly to monitor for signs of mineralocorticoid insufficiency or excess and to adjust fludrocortisone as needed (Fig. 3). This is particularly relevant when changing the GC type or dose, since different GCs have varying degrees of mineralocorticoid activity. In addition, very high 17-OHP concentrations have shown antimineralocorticoid activity in vitro (87); thus, the risk of salt wasting may lessen as disease control improves and 17-OHP decreases. If symptoms of mineralocorticoid insufficiency are present (fatigue that begins on awaking in the morning and does not improve after a dose of GC, dizziness, orthostasis, low blood pressure, salt craving, dehydration, hyponatremia, hyperkalemia, and deterioration of androgen control), consider an increase in fludrocortisone. High blood pressure, edema, puffiness, hypokalemia, or suppressed plasma renin activity can indicate mineralocorticoid excess, and a reduction in fludrocortisone should be considered (88-90).

In addition to these laboratory assessments, patients should be routinely examined for clinical signs of excess ACTH or hyperandrogenism. In female patients, this includes monitoring for menstrual irregularities and worsening acne or hirsutism. Male patients should be monitored for development or progression of TARTs and other signs of subfertility or infertility, such as azoospermia, testicular atrophy, or abnormal gonadotropin levels. Such clinical symptoms, even if androgen levels remain at goal, may indicate that the GC regimen should be adjusted or that adherence with the treatment regimen should be addressed.

The timing of GC dose reductions can vary, but it is recommended to wait at least 4 weeks between GC dose reduction steps to reduce the risk of GC withdrawal symptoms (81). If withdrawal symptoms occur, they should be resolved for at least 1 to 2 weeks before attempting another GC dose reduction. Patients who experience intolerable GC withdrawal symptoms should increase halfway back to the prior dose and attempt a smaller GC reduction ∼1 to 2 weeks after the symptoms resolve.

If there are signs of adrenal insufficiency at any point during the GC reduction process, the GC dose should be increased by ∼2.5 mg/d HCe; do not reduce the GC dose any further.

Throughout the GC reduction process, patients should continue to be counseled on the symptoms of GC withdrawal, adrenal insufficiency, and mineralocorticoid insufficiency. At each visit, patients should be provided with updated stress dosing instructions (see ***Stress Dosing***), which may change as the GC regimen shifts—for example, from “just double your GC dose” when taking 10 mg TID (∼17 mg/m^2^/d) HCe spread from morning to night to “take 10 to 20 mg hydrocortisone every 6 to 8 hours” (depending on the degree of stress) when taking 10 to 15 mg/d (∼7 mg/m^2^/d) HCe with the last dose at 1400 hours. In addition, it is critical to continue to emphasize the importance of taking prescribed GCs to prevent life-threatening adrenal crisis.

## GC withdrawal

Symptoms of GC withdrawal are nonspecific and can include headache, fatigue, body aches, joint pain, anorexia, and nausea (Fig. 3) (81). Any patient lowering their GC dose can experience GC withdrawal, but patients on higher GC doses or taking long-acting GCs, such as dexamethasone, are at higher risk. To alleviate symptoms, patients can consider engaging in light activity (eg, gentle stretching, water aerobics, walking); relaxing with warm baths, massages, or meditation; or taking nonsteroidal anti-inflammatory drugs. Symptoms usually improve or resolve within a few days to weeks following GC reduction.

## Adrenal insufficiency

Symptoms of adrenal insufficiency are similar to those of GC withdrawal but occur when GCs are decreased below that required for cortisol replacement (Fig. 3). Since the mean physiologic GC dose is ∼10 to 15 mg/d (6 to 8 mg/m^2^/d) HCe, symptoms are more likely to be due to adrenal insufficiency if the GC dose is lower than 20 mg/d HCe. Fatigue is the most common symptom of adrenal insufficiency; headache, body aches, joint pain, unintentional weight loss, and nausea can also occur. In adrenal insufficiency, hyponatremia and hypoglycemia can be observed. Symptoms of adrenal insufficiency do not improve until GC doses are increased to provide sufficient cortisol replacement.

## Stress dosing

During periods of significant physiological stress—such as febrile illness (temperature >38 °C/100.4°F), illness requiring bedrest, vomiting or diarrhea due to gastroenteritis, major trauma, or surgery requiring general anesthesia—patients with CAH should promptly increase their GC dose to prevent life-threatening adrenal crisis (2). Historically, it was common to recommend patients to double or triple their usual GC dose in times of stress (12, 91), but this practice may not provide sufficient cortisol replacement in patients who have reduced GC doses with crinecerfont. Instead, these patients should be advised to stress dose with oral hydrocortisone up to 30 to 50 mg/m^2^/d (∼50 to 100 mg/d) spread evenly throughout the day, preferably with a dose every 6 to 8 hours, or with an equivalent prednisone/prednisolone/methylprednisolone dose in 1 to 2 divided doses; dexamethasone is not appropriate for stress dosing (2). Stress dosing should be continued for 2 to 5 days until well (or for the duration of antibiotic treatment, as recommended by the European Society of Endocrinology/Endocrine Society joint guideline on GC-induced adrenal insufficiency (81)). Patients who stress dose with higher doses of hydrocortisone (greater than 40 mg/d) generally do not require extra fludrocortisone, as such doses may provide sufficient mineralocorticoid activity.

## Adrenal crisis

Adrenal crisis is a potentially fatal complication of adrenal insufficiency, characterized by acute cortisol deficiency during periods of physiological stress. Common triggers include severe illness, trauma, surgery, and gastrointestinal symptoms that impair oral medication absorption (10, 81). Clinical features typically include hypotension, hypovolemic shock, hypoglycemia, nausea, vomiting, dizziness, profound fatigue, and weakness (81).

To prevent adrenal crisis, patients with CAH must receive appropriate stress dosing of GCs during periods of increased cortisol demand (see ***Stress Dosing***). All individuals at risk should be educated on stress dosing protocols, carry medical alert identification, and have injectable hydrocortisone readily available.

Patients with CAH who present with persistent vomiting or diarrhea due to gastrointestinal illness, systemic infection requiring hospitalization or IV antibiotics (eg, sepsis), or acute trauma associated with significant blood loss or hospital admission should receive an immediate IM or IV injection of hydrocortisone 100 mg. If symptoms do not resolve or signs of hemodynamic instability develop, urgent hospital admission is warranted for parenteral GC and fluid resuscitation.

For patients requiring hospitalization, stress dosing should begin with a hydrocortisone 100 mg IV bolus or IM injection, followed by either a continuous IV infusion of 200 mg over 24 hours or, if infusion is not feasible, 50 mg IV every 6 hours (2, 81, 92). The duration and intensity of GC therapy should be individualized based on the nature of the stressor and the patient's clinical response.

## Conclusion

Given the lack of real-world guidance, we provide practical suggestions for GC tapering after initiating crinecerfont. Overall, GC reduction should be approached slowly and with caution in order to reduce the risk of the patient developing symptoms of GC withdrawal or adrenal insufficiency and to minimize the potential for adrenal crisis. Each step should be tailored to the patient's treatment goals, cortisol needs, and lifestyle preferences. We advise targeting the lowest GC dose that maintains androstenedione at goal, approximating normal circadian cortisol exposure to the extent possible, without lowering the GC dose below that needed for physiologic cortisol replacement. It is critical to provide patients with appropriate guidance for stress dosing at each visit and emphasize that taking crinecerfont does not eliminate the need to take GCs.

The availability of crinecerfont has initiated a shift in the approach to treatment for patients with classic CAH by enabling the use of lower GC doses primarily for cortisol replacement. We anticipate that our practical strategies for reducing GC doses may become increasingly relevant as the treatment paradigm for classic CAH moves toward physiologic GC replacement with adjunctive androgen control.

## Data Availability

Data sharing is not applicable to this article as no datasets were generated or analyzed during the current study.

## References

[1] Merke DP, Auchus RJ. Congenital adrenal hyperplasia due to 21-hydroxylase deficiency. N Engl J Med. 2020;383(13):1248‐1261. 32966723 10.1056/NEJMra1909786

[2] Speiser PW, Arlt W, Auchus RJ, et al Congenital adrenal hyperplasia due to steroid 21-hydroxylase deficiency: an Endocrine Society clinical practice guideline. J Clin Endocrinol Metab. 2018;103(11):4043‐4088. 30272171 10.1210/jc.2018-01865 PMC6456929

[3] Auer MK, Nordenstrom A, Lajic S, Reisch N. Congenital adrenal hyperplasia. Lancet. 2023;401(10372):227‐244. 36502822 10.1016/S0140-6736(22)01330-7

[4] Yang M, White PC. Genetics and pathophysiology of classic congenital adrenal hyperplasia due to 21-hydroxylase deficiency. J Clin Endocrinol Metab. 2025;110(Suppl 1):S1‐S12. 39836621 10.1210/clinem/dgae535 PMC11749890

[5] El-Maouche D, Arlt W, Merke DP. Congenital adrenal hyperplasia. Lancet. 2017;390(10108):2194‐2210. 28576284 10.1016/S0140-6736(17)31431-9

[6] Mallappa A, Merke DP. Management challenges and therapeutic advances in congenital adrenal hyperplasia. Nat Rev Endocrinol. 2022;18(6):337‐352. 35411073 10.1038/s41574-022-00655-w PMC8999997

[7] Claahsen-van der Grinten HL, Speiser PW, Ahmed SF, et al Congenital adrenal hyperplasia–current insights in pathophysiology, diagnostics, and management. Endocr Rev. 2022;43(1):91‐159. 33961029 10.1210/endrev/bnab016 PMC8755999

[8] Sarafoglou K, Merke DP, Reisch N, Claahsen-van der Grinten H, Falhammar H, Auchus RJ. Interpretation of steroid biomarkers in 21-hydroxylase deficiency and their use in disease management. J Clin Endocrinol Metab. 2023;108(9):2154‐2175. 36950738 10.1210/clinem/dgad134 PMC10438890

[9] Husebye ES, Pearce SH, Krone NP, Kampe O. Adrenal insufficiency. Lancet. 2021;397(10274):613‐629. 33484633 10.1016/S0140-6736(21)00136-7

[10] Bancos I, Kim H, Cheng HK, et al Glucocorticoid therapy in classic congenital adrenal hyperplasia: traditional and new treatment paradigms. Expert Rev Endocrinol Metab. 2025;20(1):33‐49. 39871142 10.1080/17446651.2025.2450423

[11] Falhammar H, Frisén L, Norrby C, et al Increased mortality in patients with congenital adrenal hyperplasia due to 21-hydroxylase deficiency. J Clin Endocrinol Metab. 2014;99(12):E2715‐E2721. 25279502 10.1210/jc.2014-2957

[12] Tschaidse L, Wimmer S, Nowotny HF, et al Frequency of stress dosing and adrenal crisis in paediatric and adult patients with congenital adrenal hyperplasia: a prospective study. Eur J Endocrinol. 2024;190(4):275‐283. 38584334 10.1093/ejendo/lvae023

[13] El-Maouche D, Hargreaves CJ, Sinaii N, Mallappa A, Veeraraghavan P, Merke DP. Longitudinal assessment of illnesses, stress dosing, and illness sequelae in patients with congenital adrenal hyperplasia. J Clin Endocrinol Metab. 2018;103(6):2336‐2345. 29584889 10.1210/jc.2018-00208 PMC6276663

[14] Rushworth RL, Torpy DJ, Falhammar H. Adrenal crisis. N Engl J Med. 2019;381(9):852‐861. 31461595 10.1056/NEJMra1807486

[15] Iezzi ML, Lasorella S, Varriale G, Zagaroli L, Ambrosi M, Verrotti A. Clitoromegaly in childhood and adolescence: behind one clinical sign, a clinical sea. Sex Dev. 2018;12(4):163‐174. 29804109 10.1159/000489385

[16] Yankovic F, Cherian A, Steven L, Mathur A, Cuckow P. Current practice in feminizing surgery for congenital adrenal hyperplasia; a specialist survey. J Pediatr Urol. 2013;9(6):1103‐1107. 23693144 10.1016/j.jpurol.2013.03.013

[17] Nokoff NJ, Buchanan C, Barker JM. Clinical manifestations and treatment challenges in infants and children with classic congenital adrenal hyperplasia due to 21-hydroxylase deficiency. J Clin Endocrinol Metab. 2025;110(Suppl 1):S13‐S24. 39836622 10.1210/clinem/dgae563 PMC11749889

[18] Bretones P, Riche B, Pichot E, et al Growth curves for congenital adrenal hyperplasia from a national retrospective cohort. J Pediatr Endocrinol Metab. 2016;29(12):1379‐1388. 27852974 10.1515/jpem-2016-0156

[19] Muthusamy K, Elamin MB, Smushkin G, et al Clinical review: adult height in patients with congenital adrenal hyperplasia: a systematic review and metaanalysis. J Clin Endocrinol Metab. 2010;95(9):4161‐4172. 20823467 10.1210/jc.2009-2616

[20] Hirschberg AL, Gidlöf S, Falhammar H, et al Reproductive and perinatal outcomes in women with congenital adrenal hyperplasia: a population-based cohort study. J Clin Endocrinol Metab. 2021;106(2):e957‐e965. 33135723 10.1210/clinem/dgaa801

[21] Astapova O, Minor BM, Hammes SR. Physiological and pathological androgen actions in the ovary. Endocrinology. 2019;160(5):1166‐1174. 30912811 10.1210/en.2019-00101 PMC6937455

[22] Pofi R, Caratti G, Ray DW, Tomlinson JW. Treating the side effects of exogenous glucocorticoids; can we separate the good from the bad? Endocr Rev. 2023;44(6):975‐1011. 37253115 10.1210/endrev/bnad016 PMC10638606

[23] Engberg H, Nordenstrom A, Hirschberg AL. Clinical manifestations and challenges in adolescent and adult females with classic congenital adrenal hyperplasia due to 21-hydroxylase deficiency. J Clin Endocrinol Metab. 2025;110(Suppl 1):S37‐S45. 39836618 10.1210/clinem/dgae696 PMC11749906

[24] Claahsen-van der Grinten HL, Stikkelbroeck N, Falhammar H, Reisch N. Management of endocrine disease: gonadal dysfunction in congenital adrenal hyperplasia. Eur J Endocrinol. 2021;184(3):R85‐R97. 33320831 10.1530/EJE-20-1093

[25] Claahsen-van der Grinten HL, Adriaansen BPH, Falhammar H. Challenges in adolescent and adult males with classic congenital adrenal hyperplasia due to 21-hydroxylase deficiency. J Clin Endocrinol Metab. 2025;110(Suppl 1):S25‐S36. 39836620 10.1210/clinem/dgae718 PMC11749911

[26] Engels M, Span PN, van Herwaarden AE, Sweep F, Stikkelbroeck N, Claahsen-van der Grinten HL. Testicular adrenal rest tumors: current insights on prevalence, characteristics, origin, and treatment. Endocr Rev. 2019;40(4):973‐987. 30882882 10.1210/er.2018-00258

[27] Eyer de Jesus L, Paz de Oliveira AP, Porto LC, Dekermacher S. Testicular adrenal rest tumors—epidemiology, diagnosis and treatment. J Pediatr Urol. 2024;20(1):77‐87. 37845103 10.1016/j.jpurol.2023.10.005

[28] Pofi R, Krone JX, Tomlinson NP, W J. Long-term health consequences of congenital adrenal hyperplasia. Clin Endocrinol (Oxf). 2024;101(4):318‐331. 37680029 10.1111/cen.14967

[29] Casteràs A, De Silva P, Rumsby G, Conway GS. Reassessing fecundity in women with classical congenital adrenal hyperplasia (CAH): normal pregnancy rate but reduced fertility rate. Clin Endocrinol (Oxf). 2009;70(6):833‐837. 19250265 10.1111/j.1365-2265.2009.03563.x

[30] Claahsen-van der Grinten HL, Otten BJ, Hermus AR, Sweep FC, Hulsbergen-van de Kaa CA. Testicular adrenal rest tumors in patients with congenital adrenal hyperplasia can cause severe testicular damage. Fertil Steril. 2008;89(3):597‐601. 17543962 10.1016/j.fertnstert.2007.03.051

[31] King TF, Lee MC, Williamson EE, Conway GS. Experience in optimizing fertility outcomes in men with congenital adrenal hyperplasia due to 21 hydroxylase deficiency. Clin Endocrinol (Oxf). 2016;84(6):830‐836. 26666213 10.1111/cen.13001

[32] Slowikowska-Hilczer J, Hirschberg AL, Claahsen-van der Grinten H, et al Fertility outcome and information on fertility issues in individuals with different forms of disorders of sex development: findings from the dsd-LIFE study. Fertil Steril. 2017;108(5):822‐831. 28923284 10.1016/j.fertnstert.2017.08.013

[33] Improda N, Barbieri F, Ciccarelli GP, Capalbo D, Salerno M. Cardiovascular health in children and adolescents with congenital adrenal hyperplasia due to 21-hydroxilase deficiency. Front Endocrinol (Lausanne). 2019;10:212. 31031703 10.3389/fendo.2019.00212 PMC6470198

[34] Torky A, Sinaii N, Jha S, et al Cardiovascular disease risk factors and metabolic morbidity in a longitudinal study of congenital adrenal hyperplasia. J Clin Endocrinol Metab. 2021;106(12):e5247‐e5257. 33677504 10.1210/clinem/dgab133 PMC8864751

[35] Mooij CF, Webb EA, Claahsen van der Grinten HL, Krone N. Cardiovascular health, growth and gonadal function in children and adolescents with congenital adrenal hyperplasia. Arch Dis Child. 2017;102(6):578‐584. 27974295 10.1136/archdischild-2016-311910

[36] Krysiak R, Claahsen-van der Grinten HL, Reisch N, Touraine P, Falhammar H. Cardiometabolic aspects of congenital adrenal hyperplasia. Endocr Rev. 2025;46(1):80‐148. 39240753 10.1210/endrev/bnae026 PMC11720181

[37] Caetano CM, Sliwinska A, Madhavan P, Grady J, Malchoff CD. Empiric determination of the daily glucocorticoid replacement dose in adrenal insufficiency. J Endocr Soc. 2020;4(11):bvaa145. 33123657 10.1210/jendso/bvaa145 PMC7575132

[38] Purnell JQ, Brandon DD, Isabelle LM, Loriaux DL, Samuels MH. Association of 24-hour cortisol production rates, cortisol-binding globulin, and plasma-free cortisol levels with body composition, leptin levels, and aging in adult men and women. J Clin Endocrinol Metab. 2004;89(1):281‐287. 14715862 10.1210/jc.2003-030440

[39] Oprea A, Bonnet NCG, Polle O, Lysy PA. Novel insights into glucocorticoid replacement therapy for pediatric and adult adrenal insufficiency. Ther Adv Endocrinol Metab. 2019;10:2042018818821294. 30746120 10.1177/2042018818821294 PMC6360643

[40] Caetano CM, Malchoff CD. Daily glucocorticoid replacement dose in adrenal insufficiency, a mini review. Front Endocrinol (Lausanne). 2022;13:897211. 35846313 10.3389/fendo.2022.897211 PMC9276933

[41] Bornstein SR, Allolio B, Arlt W, et al Diagnosis and treatment of primary adrenal insufficiency: an Endocrine Society clinical practice guideline. J Clin Endocrinol Metab. 2016;101(2):364‐389. 26760044 10.1210/jc.2015-1710 PMC4880116

[42] Arlt W, Willis DS, Wild SH, et al Health status of adults with congenital adrenal hyperplasia: a cohort study of 203 patients. J Clin Endocrinol Metab. 2010;95(11):5110‐5121. 20719839 10.1210/jc.2010-0917 PMC3066446

[43] Falhammar H, Filipsson Nystrom H, Wedell A, Thoren M. Cardiovascular risk, metabolic profile, and body composition in adult males with congenital adrenal hyperplasia due to 21-hydroxylase deficiency. Eur J Endocrinol. 2011;164(2):285‐293. 21098686 10.1530/EJE-10-0877

[44] Falhammar H, Frisen L, Hirschberg AL, et al Increased cardiovascular and metabolic morbidity in patients with 21-hydroxylase deficiency: a Swedish population-based national cohort study. J Clin Endocrinol Metab. 2015;100(9):3520‐3528. 26126207 10.1210/JC.2015-2093

[45] Kim JH, Choi S, Lee YA, Lee J, Kim SG. Epidemiology and long-term adverse outcomes in Korean patients with congenital adrenal hyperplasia: a nationwide study. Endocrinol Metab (Seoul). 2022;37(1):138‐147. 35255606 10.3803/EnM.2021.1328 PMC8901972

[46] Li L, Bensing S, Falhammar H. Rate of fracture in patients with glucocorticoid replacement therapy: a systematic review and meta-analysis. Endocrine. 2021;74(1):29‐37. 33846948 10.1007/s12020-021-02723-z

[47] Rangaswamaiah S, Gangathimmaiah V, Nordenstrom A, Falhammar H. Bone mineral density in adults with congenital adrenal hyperplasia: a systematic review and meta-analysis. Front Endocrinol (Lausanne). 2020;11:493. 32903805 10.3389/fendo.2020.00493 PMC7438951

[48] Falhammar H, Frisen L, Hirschberg AL, Nordenskjold A, Almqvist C, Nordenstrom A. Increased prevalence of fractures in congenital adrenal hyperplasia: a Swedish population-based national cohort study. J Clin Endocrinol Metab. 2022;107(2):e475‐e486. 34601607 10.1210/clinem/dgab712 PMC8764334

[49] Chotiyarnwong P, McCloskey EV. Pathogenesis of glucocorticoid-induced osteoporosis and options for treatment. Nat Rev Endocrinol. 2020;16(8):437‐447. 32286516 10.1038/s41574-020-0341-0

[50] Jaaskelainen J, Voutilainen R. Bone mineral density in relation to glucocorticoid substitution therapy in adult patients with 21-hydroxylase deficiency. Clin Endocrinol (Oxf). 1996;45(6):707‐713. 9039336 10.1046/j.1365-2265.1996.8620871.x

[51] Schulz J, Frey KR, Cooper MS, et al Reduction in daily hydrocortisone dose improves bone health in primary adrenal insufficiency. Eur J Endocrinol. 2016;174(4):531‐538. 26811406 10.1530/EJE-15-1096

[52] Hagenfeldt K, Martin Ritzen E, Ringertz H, Helleday J, Carlstrom K. Bone mass and body composition of adult women with congenital virilizing 21-hydroxylase deficiency after glucocorticoid treatment since infancy. Eur J Endocrinol. 2000;143(5):667‐671. 11078991 10.1530/eje.0.1430667

[53] Zimmermann A, Sido PG, Schulze E, et al Bone mineral density and bone turnover in Romanian children and young adults with classical 21-hydroxylase deficiency are influenced by glucocorticoid replacement therapy. Clin Endocrinol (Oxf). 2009;71(4):477‐484. 19170706 10.1111/j.1365-2265.2008.03518.x

[54] Filipsson H, Monson JP, Koltowska-Haggstrom M, Mattsson A, Johannsson G. The impact of glucocorticoid replacement regimens on metabolic outcome and comorbidity in hypopituitary patients. J Clin Endocrinol Metab. 2006;91(10):3954‐3961. 16895963 10.1210/jc.2006-0524

[55] Falhammar H, Filipsson H, Holmdahl G, et al Fractures and bone mineral density in adult women with 21-hydroxylase deficiency. J Clin Endocrinol Metab. 2007;92(12):4643‐4649. 17878254 10.1210/jc.2007-0744

[56] Falhammar H, Filipsson Nystrom H, Wedell A, Brismar K, Thoren M. Bone mineral density, bone markers, and fractures in adult males with congenital adrenal hyperplasia. Eur J Endocrinol. 2013;168(3):331‐341. 23211577 10.1530/EJE-12-0865

[57] Sharma V, Coope H, Maskin K, et al Adverse events associated with supraphysiological glucocorticoid dosing in congenital adrenal hyperplasia (CAH): results of a structured literature review. *Endocrine Abstracts.* 2022;81:EP70.

[58] Han TS, Stimson RH, Rees DA, et al Glucocorticoid treatment regimen and health outcomes in adults with congenital adrenal hyperplasia. Clin Endocrinol (Oxf). 2013;78(2):197‐203. 22998134 10.1111/cen.12045

[59] Volkl TM, Ohl L, Rauh M, Schofl C, Dorr HG. Adrenarche and puberty in children with classic congenital adrenal hyperplasia due to 21-hydroxylase deficiency. Horm Res Paediatr. 2011;76(6):400‐410. 22123283 10.1159/000333696

[60] Kim M, Barnes C, Charlton W. Association between glucocorticoid dose with BMI and glucocorticoid-related comorbidities: data from tildacerfont phase 2a trials in classic congenital adrenal hyperplasia. J ASEAN Fed Endocr Soc. 2022;37(2):13.

[61] Borges JH, Santoro RI, de Oliveira DM, et al Cardiovascular dysfunction risk in young adults with congenital adrenal hyperplasia caused by 21-hydroxylase enzyme deficiency. Int J Clin Pract. 2021;75(7):e14233. 33884716 10.1111/ijcp.14233

[62] Tamhane S, Rodriguez-Gutierrez R, Iqbal AM, et al Cardiovascular and metabolic outcomes in congenital adrenal hyperplasia: a systematic review and meta-analysis. J Clin Endocrinol Metab. 2018;103(11):4097‐4103. 30272185 10.1210/jc.2018-01862

[63] Paizoni L, Auer MK, Schmidt H, Hubner A, Bidlingmaier M, Reisch N. Effect of androgen excess and glucocorticoid exposure on metabolic risk profiles in patients with congenital adrenal hyperplasia due to 21-hydroxylase deficiency. J Steroid Biochem Mol Biol. 2020;197:105540. 31730799 10.1016/j.jsbmb.2019.105540

[64] Whittle E, Falhammar H. Glucocorticoid regimens in the treatment of congenital adrenal hyperplasia: a systematic review and meta-analysis. J Endocr Soc. 2019;3(6):1227‐1245. 31187081 10.1210/js.2019-00136 PMC6546346

[65] Oksnes M, Bjornsdottir S, Isaksson M, et al Continuous subcutaneous hydrocortisone infusion versus oral hydrocortisone replacement for treatment of Addison's disease: a randomized clinical trial. J Clin Endocrinol Metab. 2014;99(5):1665‐1674. 24517155 10.1210/jc.2013-4253

[66] Mallappa A, Nella AA, Sinaii N, et al Long-term use of continuous subcutaneous hydrocortisone infusion therapy in patients with congenital adrenal hyperplasia. Clin Endocrinol (Oxf). 2018;89(4):399‐407. 30003563 10.1111/cen.13813 PMC6166869

[67] Mortensen ML, Ornstrup MJ, Gravholt CH. Patients with hypocortisolism treated with continuous subcutaneous hydrocortisone infusion (CSHI): an option for poorly controlled patients. Int J Endocrinol. 2023;2023:5315059. 36994228 10.1155/2023/5315059 PMC10042637

[68] Isidori AM, Venneri MA, Graziadio C, et al Effect of once-daily, modified-release hydrocortisone versus standard glucocorticoid therapy on metabolism and innate immunity in patients with adrenal insufficiency (DREAM): a single-blind, randomised controlled trial. Lancet Diabetes Endocrinol. 2018;6(3):173‐185. 29229498 10.1016/S2213-8587(17)30398-4

[69] Merke DP, Mallappa A, Arlt W, et al Modified-release hydrocortisone in congenital adrenal hyperplasia. J Clin Endocrinol Metab. 2021;106(5):e2063‐e2077. 33527139 10.1210/clinem/dgab051 PMC8063257

[70] Sarafoglou K, Auchus RJ. Future directions in the management of classic congenital adrenal hyperplasia due to 21-hydroxylase deficiency. J Clin Endocrinol Metab. 2025;110(Suppl 1):S74‐S87. 39836617 10.1210/clinem/dgae759 PMC11749912

[71] Auchus RJ, Sarafoglou K, Fechner PY, et al The effects of crinecerfont (NBI-74788), a novel CRF_1_ receptor antagonist, on adrenal androgens and precursors in patients with classic congenital adrenal hyperplasia: results from a multiple-dose phase 2 study. J Endocr Soc. 2020;4(Suppl 1):OR25‐OR03.

[72] Newfield RS, Sarafoglou K, Fechner PY, et al Crinecerfont, a CRF_1_ receptor antagonist, lowers adrenal androgens in adolescents with congenital adrenal hyperplasia. J Clin Endocrinol Metab. 2023;108(11):2871‐2878. 37216921 10.1210/clinem/dgad270 PMC10583973

[73] Auchus RJ, Hamidi O, Pivonello R, et al Phase 3 trial of crinecerfont in adult congenital adrenal hyperplasia. N Engl J Med. 2024;391(6):504‐514. 38828955 10.1056/NEJMoa2404656 PMC11309900

[74] Auchus RJ, Hamidi O, Liu C-Y, et al *Crinecerfont maintains adrenocorticotropic hormone and 17-hydroxyprogesterone levels with reduced glucocorticoid doses in adults with classic congenital adrenal hyperplasia: 1-year results from the* CAHtalyst™ Adult study. *J Endocr Soc.* 2025;9(Suppl 1):bvaf149.387.

[75] Sarafoglou K, Kim MS, Lodish M, et al Phase 3 trial of crinecerfont in pediatric congenital adrenal hyperplasia. N Engl J Med. 2024;391(6):493‐503. 38828945 10.1056/NEJMoa2404655

[76] Geffner ME, Newfield RS, Hsu Y, et al Crinecerfont reduces plasma adrenocorticotropic hormone and serum 17-hydroxyprogesterone levels in children and adolescents with classic congenital adrenal hyperplasia: 1-year results from the CAHtalyst™ Pediatric study. *J Endocr Soc.* 2025;9(Suppl 1):bvaf149.404.

[77] Srirangalingam U, Velusamy A, Binderup ML, et al Safety and pharmacokinetics of anti-ACTH antibody Lu AG13909 in patients with classic congenital adrenal hyperplasia: phase 1 open-label, multiple-ascending-dose trial protocol. J Endocr Soc. 2024;5(Suppl 1):A152‐A153.

[78] Auchus RJ, Trainer PJ, Lucas KJ, et al Once daily oral atumelnant (CRN04894) induces rapid and profound reductions of androstenedione and 17-hydroxyprogesterone in participants with classical congenital adrenal hyperplasia: initial results from a 12-week, phase 2, open-label study. J Endocr Soc. 2024;8(Suppl 1):A134.

[79] El-Maouche D, Merke DP, Vogiatzi MG, et al A phase 2, multicenter study of nevanimibe for the treatment of congenital adrenal hyperplasia. J Clin Endocrinol Metab. 2020;105(8):2771‐2778. 32589738 10.1210/clinem/dgaa381 PMC7331874

[80] Neurocrine Biosciences, Inc . CRENESSITY^®^(Crinecerfont) Capsules and CRENESSITY^®^ (Crinecerfont) Oral Solution Full Prescribing Information. Neurocrine Biosciences, Inc.; 2024.

[81] Beuschlein F, Else T, Bancos I, et al European Society of Endocrinology and Endocrine Society joint clinical guideline: diagnosis and therapy of glucocorticoid-induced adrenal insufficiency. J Clin Endocrinol Metab. 2024;190(5):G25‐G51. 10.1093/ejendo/lvae029 38714321

[82] Dauber A, Kellogg M, Majzoub JA. Monitoring of therapy in congenital adrenal hyperplasia. Clin Chem. 2010;56(8):1245‐1251. 20558634 10.1373/clinchem.2010.146035

[83] Weitzman ED, Fukushima D, Nogeire C, Roffwarg H, Gallagher TF, Hellman L. Twenty-four hour pattern of the episodic secretion of cortisol in normal subjects. J Clin Endocrinol Metab. 1971;33(1):14‐22. 4326799 10.1210/jcem-33-1-14

[84] Pruessner JC, Wolf OT, Hellhammer DH, et al Free cortisol levels after awakening: a reliable biological marker for the assessment of adrenocortical activity. Life Sci. 1997;61(26):2539‐2549. 9416776 10.1016/s0024-3205(97)01008-4

[85] Rivkees SA, Crawford JD. Dexamethasone treatment of virilizing congenital adrenal hyperplasia: the ability to achieve normal growth. Pediatrics. 2000;106(4):767‐773. 11015521 10.1542/peds.106.4.767

[86] Wilkins L . The Diagnosis and Treatment of Endocrine Disorders in Childhood and Adolescence. 1st ed. Thomas; 1950.

[87] Mooij CF, Parajes S, Pijnenburg-Kleizen KJ, Arlt W, Krone N, Claahsen-van der Grinten HL. Influence of 17-hydroxyprogesterone, progesterone and sex steroids on mineralocorticoid receptor transactivation in congenital adrenal hyperplasia. Horm Res Paediatr. 2015;83(6): 414‐421. 10.1159/000374112 25896481

[88] Lang K, Quinkler M, Kienitz T. Mineralocorticoid replacement therapy in salt-wasting congenital adrenal hyperplasia. Clin Endocrinol (Oxf). 2024;101(4):346‐358. 37564007 10.1111/cen.14959

[89] Reisch N, Arlt W, Krone N. Health problems in congenital adrenal hyperplasia due to 21-hydroxylase deficiency. Horm Res Paediatr. 2011;76(2):73‐85. 21597280 10.1159/000327794

[90] Bonfig W, Roehl FW, Riedl S, et al Blood pressure in a large cohort of children and adolescents with classic adrenal hyperplasia (CAH) due to 21-hydroxylase deficiency. Am J Hypertens. 2016;29(2):266‐272. 26071487 10.1093/ajh/hpv087

[91] Witchel SF, Miller T, McCann E, Gupta A. Life with classic congenital adrenal hyperplasia due to 21-hydroxylase deficiency: challenges and burdens. J Clin Endocrinol Metab. 2025;110(Suppl 1):S56‐S66. 39836616 10.1210/clinem/dgae728 PMC11749882

[92] Pazderska A, Pearce SH. Adrenal insufficiency—recognition and management. Clin Med (Lond). 2017;17(3):258‐262. 28572228 10.7861/clinmedicine.17-3-258 PMC6297573

